# *Kappaphycus alvarezii* as a Food Supplement Prevents Diet-Induced Metabolic Syndrome in Rats

**DOI:** 10.3390/nu9111261

**Published:** 2017-11-17

**Authors:** Stephen Wanyonyi, Ryan du Preez, Lindsay Brown, Nicholas A. Paul, Sunil K. Panchal

**Affiliations:** 1 Functional Foods Research Group, Institute for Agriculture and the Environment, University of Southern Queensland, Toowoomba, QLD 4350, Australia; Stephen.Wanyonyi@usq.edu.au (S.W.); Ryan.duPreez@usq.edu.au (R.d.P.); Lindsay.Brown@usq.edu.au (L.B.); 2 School of Health and Wellbeing, University of Southern Queensland, Toowoomba, QLD 4350, Australia; 3 Faculty of Science, Health, Education and Engineering, University of the Sunshine Coast, Maroochydore, QLD 4558, Australia; npaul@usc.edu.au

**Keywords:** metabolic syndrome, red seaweed, obesity, inflammation, carrageenan, *Kappaphycus alvarezii*, potassium, salt

## Abstract

The red seaweed, *Kappaphycus alvarezii*, was evaluated for its potential to prevent signs of metabolic syndrome through use as a whole food supplement. Major biochemical components of dried *Kappaphycus* are carrageenan (soluble fiber ~34.6%) and salt (predominantly potassium (K) 20%) with a low overall energy content for whole seaweed. Eight to nine week old male Wistar rats were randomly divided into three groups and fed for 8 weeks on a corn starch diet, a high-carbohydrate, high-fat (H) diet, alone or supplemented with a 5% (*w*/*w*) dried and milled *Kappaphycus* blended into the base diet. H-fed rats showed symptoms of metabolic syndrome including increased body weight, total fat mass, systolic blood pressure, left ventricular collagen deposition, plasma triglycerides, and plasma non-esterified fatty acids along with fatty liver. Relative to these obese rats, *Kappaphycus*-treated rats showed normalized body weight and adiposity, lower systolic blood pressure, improved heart and liver structure, and lower plasma lipids, even in presence of H diet. *Kappaphycus* modulated the balance between Firmicutes and Bacteroidetes in the gut, which could serve as the potential mechanism for improved metabolic variables; this was accompanied by no damage to the gut structure. Thus, whole *Kappaphycus* improved cardiovascular, liver, and metabolic parameters in obese rats.

## 1. Introduction

Regular consumption of seaweeds in Japan, Korea, and China occurs together with a relatively low incidence of cardiovascular and metabolic disorders relative to Western countries [1,2,3,4]. In Japan, for example, more than 20 species of seaweeds are regularly included in meals and each have different biochemical properties and herbal uses [5]. This diversity of seaweeds in the diet suggests that supplementation of Western diets with seaweeds should be investigated as an intervention to reduce the incidence of cardiovascular disease and diabetes.

Edible seaweeds contain a large variety of phylum-specific dietary fiber including alginates, fucans, and laminarans from brown seaweeds (Phylum Ochrophyta, Class Phaeophyceae), galactans, agar, and carrageenans from red seaweeds (Phylum Rhodophyta), and ulvans from green seaweeds (Phylum Chlorophyta) [6,7,8]. A modest 8 g/day serving of seaweed could provide up to 12.5% of the recommended daily intake of dietary fiber [9]. Additionally, seaweeds contain varying amounts of protein, with some red seaweed species containing up to 26.6% proteins [10], so that dietary seaweeds confer the advantage of low energy density [9]. Most of the seaweeds that are staple foods in Japan, China, and Korea are cold-water genera of brown (*Saccharina*/*Laminaria*—kombu, *Undaria*—wakame, *Sargassum*—hijiki) or red seaweeds (*Porphyra*/*Pyropia* species—nori). These species have dominated the anti-obesity studies to date, with far less known about the main species of tropical red seaweeds that are now being cultured on a large scale in Indonesia and the Philippines. A recent study with one such seaweed, *Gracilaria*, revealed its potential as an intervention against metabolic diseases [11]. In streptozotocin-induced diabetic mice, polysaccharides from *Gracilaria lemaneiformis* were more effective at regulating the blood insulin concentrations, lipid parameters, and blood urea nitrogen than metformin [11]. However, our understanding of the potential of red seaweed commodities including *Kappaphycus alvarezii* (hereafter *Kappaphycus*) as functional foods remains limited.

Functional foods such as red seaweeds could serve as potential therapeutic options for metabolic syndrome [12], a constellation of risk factors for type 2 diabetes and cardiovascular disease. There is some evidence for antioxidant and blood lipid-lowering properties of *Kappaphycus* [13,14]. Furthermore, an in vitro study using the extracted sulfated fractions of *κ*-carrageenan from *Kappaphycus* demonstrated the potential of this seaweed as an intervention for colon cancer [15]. However, the major problem with the studies to date on *Kappaphycus* is that the focus has been almost exclusively on the use of extracted carrageenan for its effects. Thus, this study looked at the whole *Kappaphycus* as the intervention, acknowledging that the dried seaweed product is more than a source of carrageenans, and the investigation of this functional food requires use of the whole seaweed. Further, isolated carrageenan has been used to induce paw edema to test anti-inflammatory compounds [16,17,18], which is contradictory to the hypothesis of this study. As carrageenan is a soluble fiber, it has potential to slow down digestion [9] and hence help in improving metabolism.

In this study, we have evaluated the potential of *Kappaphycus* as a whole food to attenuate the development of obesity in high-carbohydrate, high-fat diet-fed rats that mimic symptoms of human metabolic syndrome including central obesity, hypertension, dyslipidemia, and impaired glucose tolerance together with the cardiovascular and liver complications of metabolic syndrome [19]. We evaluated the responses to *Kappaphycus* in a prevention protocol where rats were given high-carbohydrate, high-fat diet with *Kappaphycus* for a total of 8 weeks. Additionally, we investigated the effect of supplementation with *Kappaphycus* on the balance of gut microbiota as a potential mechanism for improving metabolic syndrome.

## 2. Materials and Methods

### 2.1. Red Seaweed Source

Air-dried samples of *Kappaphycus* were collected from a seaweed trader in Suva, Fiji, in June 2015. The source of *Kappaphycus* was shallow water farms located at Yaqeta village in the Yasawa Islands, Fiji. Two bulk samples of ~3 kg each, comprising multiple individual thalli, were packed in vacuum-sealed bags containing silica desiccant and transported to Australia.

### 2.2. Seaweed Analysis

The two 3 kg samples of *Kappaphycus* were dried to <2% moisture content and subsequently milled using a blender prior to further analyses. A total of 1 kg of each replicate sample was combined, homogenized, and stored at −20 °C prior to use in the rat study. The biochemical profile of each 3 kg milled seaweed sample was analyzed separately for its proximate composition (dietary fiber, protein, lipid, ash, and energy content) and elemental composition (carbon (C), hydrogen (H), oxygen (O), nitrogen (N), sulfur (S), metals, metalloids, and halogens).

For the proximate analyses, total dietary fiber content, including soluble and insoluble components, was analyzed by Grain Growers Ltd. (Sydney, NSW, Australia) on a 10 g sub-sample following standard methods (Association of Official Analytical Chemists (AOAC) Official Method 985.29 total dietary fiber in foods, and AOAC Official Method 993.19 soluble dietary fiber in food and food products). Protein content was determined as the sum of amino acids based on quantitative amino acid analysis performed at The Australian Proteome Analysis Facility [20]. Ash content was quantified by combustion in air (550 °C, 6 h) (SEM muffle furnace, LabTek, Brendale, QLD, Australia). Lipid content was quantified on a 200 mg sub-sample using solvent extraction [21,22]. Moisture content was measured on a 1 g biomass at 105 °C to constant weight (MS-70 moisture analyzer, A & D Company Ltd., Thebarton, SA, Australia). Carbohydrates were calculated by difference as 100 − Σ (lipid, protein, ash, moisture) where lipid, protein, ash, and moisture contents were expressed as a percentage of the original biomass. The higher heating value (HHV) or energy content (kJ/g) of *Kappaphycus* was calculated based on ash content and C, H, O, N and S elemental composition [23].

A sub-sample (200 mg) of each replicate was analyzed for the content of C, H, O, N, and S as well as Cl, Br, F, and I (OEA Laboratory Ltd., Cornwall, UK). The content of metals and metalloids (22 elements) of the seaweed was measured by Inductively Coupled Plasma Mass Spectrometry (ICP/MS with Varian 820-MS, Mulgrave, VIC, Australia) at the Advanced Analytical Centre of James Cook University, Townsville, Australia.

### 2.3. Rats and Diets

All experimental protocols were approved by the Animal Ethics Committee of the University of Southern Queensland (approval number 15REA007) under the guidelines of the National Health and Medical Research Council of Australia. The experimental protocol consisted of 30 male Wistar rats (8–9 weeks old; 330–335 g) purchased from the Animal Resource Centre, Murdoch, WA, Australia. Rats were randomly divided into three experimental groups each consisting of ten rats. One group was fed on corn starch diet (C) which did not induce any complications of metabolic syndrome. The second group was fed with a high-carbohydrate, high-fat diet (H) to induce metabolic syndrome. The third group was fed with high-carbohydrate, high-fat diet supplemented with dried milled *Kappaphycus* (HR, 5% of the food). All groups were fed for 8 weeks on their respective diets.

All rats were housed in individual cages in a temperature-controlled (21 ± 2 °C) room with an automated 12-h light/dark cycle environment and *ad libitum* access to food and water. C diet was prepared by thoroughly mixing 570 g corn starch, 155 g powdered rat food, 25 g Hubbel, Mendel, and Wakeman salt mixture (MP Biomedicals, Seven Hills, NSW, Australia), and 250 g water per kilogram of food. H diet was prepared by thoroughly mixing 175 g fructose, 395 g condensed milk, 200 g beef tallow, 155 g powdered rat food, 25 g Hubbel, Mendel, and Wakeman salt mixture, and 50 g water per kilogram of food [19]. HR diet was prepared by replacing water with dried milled *Kappaphycus*. C rats were given normal drinking water whereas H and HR rats were given 25% fructose (*w*/*v*) in drinking water. The energy content of the diet and feed conversion efficiency were calculated [19]. Rats were measured daily for their body weights and intakes of food and water.

### 2.4. Systolic Blood Pressure

Systolic blood pressure was measured under light sedation by intraperitoneal injection with Zoletil (tiletamine 10 mg/kg, zolazepam 10 mg/kg; Virbac, Peakhurst, NSW, Australia) [19].

### 2.5. Oral Glucose Tolerance Test

Oral glucose tolerance test was performed on rats after overnight (~12 h) food deprivation [19]. Briefly, basal blood glucose concentrations were determined in tail vein blood using Medisense Precision Q.I.D. glucometer (Abbott Laboratories, Bedford, MA, USA). After the initial blood glucose measurements, rats were given 2 g/kg body weight of glucose as a 40% (*w*/*v*) aqueous glucose solution via oral gavage. Tail vein blood samples were then taken 30, 60, 90, and 120 min after glucose administration [19]. During the food-deprivation period, fructose-supplemented drinking water in H and HR rats was replaced with normal drinking water.

### 2.6. Body Composition

Dual-energy *X*-ray absorptiometry was performed on all rats at the end of protocol using a Norland XR36 DXA instrument (Norland Corp., Fort Atkinson, WI, USA) [19]. Briefly, rats were anesthetized by intraperitoneal injection of Zoletil (tiletamine 10 mg/kg and zolazepam 10 mg/kg) and Ilium Xylazil (xylazine 6 mg/kg; Troy Laboratories, Smithfield, NSW, Australia) [19]. Visceral adiposity index (%) was calculated [24].

### 2.7. Organ Weights

Terminal euthanasia was induced by intraperitoneal injection of Lethabarb (pentobarbitone sodium, 100 mg/kg; Virbac, Peakhurst, NSW, Australia) and ~6 mL blood was immediately drawn from the abdominal aorta and processed for plasma collection [19]. Hearts were separated into right ventricle and left ventricle with septum for weighing. Livers and abdominal fat pads (retroperitoneal, epididymal, and omental) were isolated and weighed. Organ weights were normalized to the tibial length at the time of isolation and the final organ weights were presented in mg of tissue/mm of tibial length [19].

### 2.8. Histology

Hearts and livers from rats in each group were collected and fixed in 10% neutral buffered formalin for 3 days. Tissues were then processed and blocked in wax before thin sections (5 μm) were cut and placed on slides. Heart tissues were stained with hematoxylin and eosin or picrosirius red. Liver, ileum, and colon sections were stained with hematoxylin and eosin. Images were taken using an EVOS FL Colour Imaging System (v 1.4 (Rev 26059); Advanced Microscopy Group, Bothell, WA, USA).

### 2.9. Biochemical Analyses of Rat Plasma

Plasma activities of alanine transaminase and aspartate transaminase, and plasma concentrations of total cholesterol, triglycerides, non-esterified fatty acids, and potassium (K) were determined [19].

### 2.10. Fecal Lipid Measurements

Fecal lipids were extracted using Folch method [21]. Briefly, 1 g of freshly collected feces were air dried and added to a homogenization tube containing 5 mL saline. The pellets were homogenized to form a suspension. The suspension was thoroughly mixed with an equal volume of chloroform in methanol (2:1, *v*/*v*) and centrifuged at 1000× g for 10 min at room temperature. After centrifugation, the lower liquid phase was isolated and transferred to a separate vial. The extracted lipids were air-dried and weighed.

### 2.11. Gut Microbiota Diversity Profiling

Immediately following euthanasia and organ removal, two or three fecal pellets were collected from the colon of rats and stored at −80 °C in nuclease-free tubes. DNA extraction and diversity profiling were performed by the Australian Genome Research Facility, Brisbane, QLD, Australia. The V3-V4 region of the 16S rRNA gene was selected for amplification. The primers used were F341 (5′-CCTAYGGGRBGCASCAG-3′) and R806 (5′-GGACTACNNGGGTATCTAAT-3′). PCR amplicons were generated using AmpliTaq Gold 360 mastermix (Life Technologies, Scoresby, VIC, Australia) for the primary PCR. A secondary PCR to index the amplicons was performed with TaKaRa Taq DNA Polymerase (Clontech, Mountain View, CA, USA). The resulting amplicons were measured by fluorometry (Invitrogen Picogreen, Mount Waverley, VIC, Australia) and normalized. The equimolar pool was then measured by qPCR (KAPA) followed by sequencing on the Illumina MiSeq (Illumina Inc., San Diego, CA, USA) with 2 × 300 base pairs paired-end chemistry.

Paired-ends reads were assembled by aligning the forward and reverse reads using PEAR (version 0.9.5, The Exelixis Lab, Heidelberg, Germany) [25]. Primers were identified and trimmed. Trimmed sequences were processed using Quantitative Insights into Microbial Ecology (QIIME 1.8) [26] USEARCH (version 7.1.1090) [27,28] and UPARSE software [29]. Using USEARCH, sequences were quality filtered, full length duplicate sequences were removed and sorted by abundance. Singletons or unique reads in the data set were discarded. Sequences were clustered followed by chimera filtered using “rdp_gold” database as the reference. To obtain the number of reads in each Operational Taxonomic Unit (OTU), reads were mapped back to OTUs with a minimum identity of 97%. Using QIIME, taxonomy was assigned using Greengenes database (version 13_8, August 2013) [30]. A heat map was constructed using R statistical software according to the developer’s instructions to visualize the relative abundance of each bacterial species and their respective phyla.

### 2.12. Metal and Metalloid Liver Analyses

Livers were dissected out and dried at 60 °C for 24 h. A suite of 22 elements was analyzed by the Advanced Analytical Centre at James Cook University using Inductively Coupled Plasma Mass Spectrometer.

### 2.13. Statistical Analysis

All data are presented as mean ± standard error of the mean (SEM). Differences between the groups were determined by one-way analysis of variance. Statistically significant variables were treated with Neumann-Keuls *post hoc* test to compare all groups of rats. Statistical analyses were performed using GraphPad Prism version 5 for Windows (San Diego, CA, USA). *P* values of <0.05 were considered statistically significant.

## 3. Results

### 3.1. Composition of Kappaphycus

Dried *Kappaphycus* contained carbohydrates (38.3% dry weight (d.w.)) and minerals (ash content: 58.0% d.w.) with relatively small amount of proteins (1.34% d.w.; sum of amino acids, Supplementary Table S1) and lipids (0.62% d.w.) and a low energy content of 5.23 kJ/g. The carbohydrate component of the seaweed was further characterized into insoluble and soluble dietary fiber, of which the soluble fiber component comprised 34.6% d.w. of the whole biomass, indicating that 90% of the carbohydrate is soluble fiber.

The mineral content included potassium (K, 20% d.w.) and sodium (Na, 3.7% d.w.), primarily as chloride salts (Cl, 23% d.w.), and the ratio of sodium:potassium (Na:K) was 0.19. There were relatively low concentrations of halogens, for example, bromine (Br) was 0.12% d.w. and iodine (I) was not detected (Supplementary Table S2). A range of other metal and metalloid elements was present, including calcium (Ca, 28.96 g/kg) and magnesium (Mg, 5.69 g/kg), as well as other trace elements including iron (Fe), boron (B), and manganese (Mn) (Supplementary Table S3).

### 3.2. Diet Intake and Body Composition

At the end of the protocol, body weight and body mass index of H rats were higher than C rats while HR rats had similar body weight and body mass index to C rats (Table 1). Food intake was higher in C rats relative to H and HR rats while energy intake was higher in H and HR rats compared to C rats (Table 1). There were no differences in water intakes between C, H, and HR rats. Feed conversion efficiency was higher in H rats than in C and HR rats (Table 1). H rats had higher abdominal circumference than C rats whereas HR rats had lower abdominal circumference compared to H rats (Table 1). Retroperitoneal, omental, and total abdominal fat were higher in H rats compared to C and HR rats whereas epididymal fat was not different between these groups (Table 1). Similar to total abdominal fat content, total body fat was higher in H rats than in C and HR rats whereas lean mass was similar in C and H rats and lower in HR rats than in C and H rats (Table 1). Bone mineral content and bone mineral density were higher in H rats compared to C rats while these parameters were lower in HR rats compared to H rats (Table 1).

### 3.3. Oral Glucose Tolerance, Plasma Biochemistry, and Fecal Lipids

Basal blood glucose concentrations and area under the curve at 8 weeks were higher in H rats compared to C rats (Table 1). Although basal blood glucose concentrations at 8 weeks were lower in HR rats than in H rats, the areas under the curve were similar in H and HR rats (Table 1). Plasma total cholesterol was unchanged between the groups whereas plasma triglycerides and non-esterified fatty acids were in the order of C < HR < H (Table 1). However, total cholesterol concentrations were not different. HR diets decreased plasma non-esterified fatty acids and triglycerides (Table 1). Fecal lipids were higher in H rats compared to C rats where HR had lower fecal lipids than H rats (Table 1). Plasma potassium concentrations were lower in H rats compared to C rats whereas the HR rats had higher plasma potassium concentrations than H rats (Table 1).

### 3.4. Cardiovascular Structure and Function

**Figure 1:**
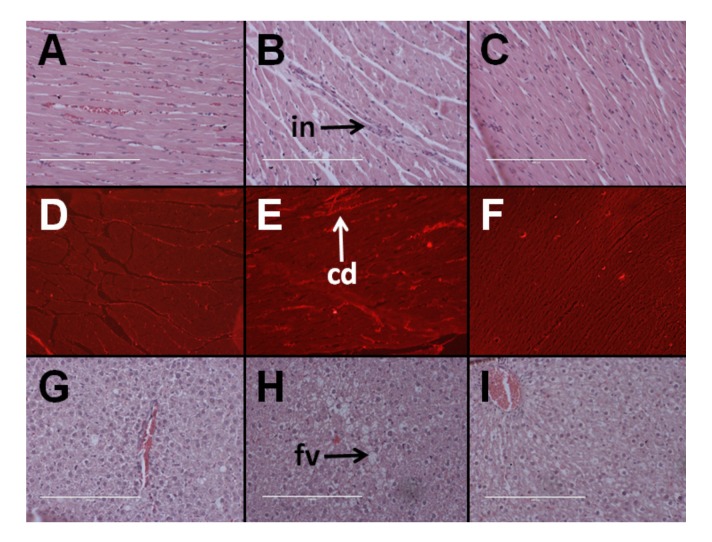
Effects of *Kappaphycus* on inflammation (top row—“in”) and collagen deposition (middle row—“cd”) in the heart using hematoxylin and eosin stain and picrosirius red stain, respectively, in C rats (**A**,**D**), H rats (**B**,**E**), and HR rats (**C**,**F**). Effects of *Kappaphycus* on inflammation and fat deposition (bottom row—“fv”) in the liver using hematoxylin and eosin stain in C rats (**G**), H rats (**H**), and HR rats (**I**).

At 8 weeks, systolic blood pressure was higher in H rats than C rats. *Kappaphycus* suppressed the increase in blood pressure in HR rats in comparison to H rats; however, the systolic blood pressure was higher in HR rats than C rats. There were no differences in LV + septum wet weight and RV wet weight between the groups (Table 1). H rats had higher infiltration of inflammatory cells and collagen deposition in the left ventricle than in C rats whereas HR rats had lower infiltration of inflammatory cells and collagen deposition than H rats (Figure 1).

### 3.5. Hepatic Structure and Function

Hematoxylin and eosin staining of liver sections from C rats showed that there was minimal fat deposition in the livers of C rats and infiltration by inflammatory cells was not observed (Figure 1G). H rats showed the presence of fat vacuoles and infiltration of inflammatory cells (Figure 1H). HR rats had lower fat deposition in the liver than H rats with very little to no inflammation (Figure 1I). Plasma activities of AST were similar between all the groups. Plasma ALT activities were higher in HR rats than in C and H rats (Table 1).

Metal and metalloid liver analyses revealed widespread decreases in the elemental content of liver of HR rats relative to both C and H rats, with only some minor increases in particular HR vs. C and HR vs. H comparisons (Table 2). As the most prominent examples in comparison to H rats (>1/3 reduction), *Kappaphycus* decreased the concentrations in HR rats of barium (Ba) (to 28% of H level), aluminium (Al) (48%), molybdenum (Mo) (52%), strontium (Sr) (53%), arsenic (As) (62%), and zinc (Zn) (65%). Similarly, in comparison to C rats, arsenic (As) and molybdenum (Mo) decreased to 29% and 64%, respectively, in HR rats.

While there was more potassium (K) in the HR diets (due to the 5% seaweed portion delivering ~10× more potassium (K) to the overall diet), there was a lower concentration of potassium (K) in the liver (HR resulted in 71% of H and HR was 69% of C).

### 3.6. Gut Structure and Microbiota

**Figure 2:**
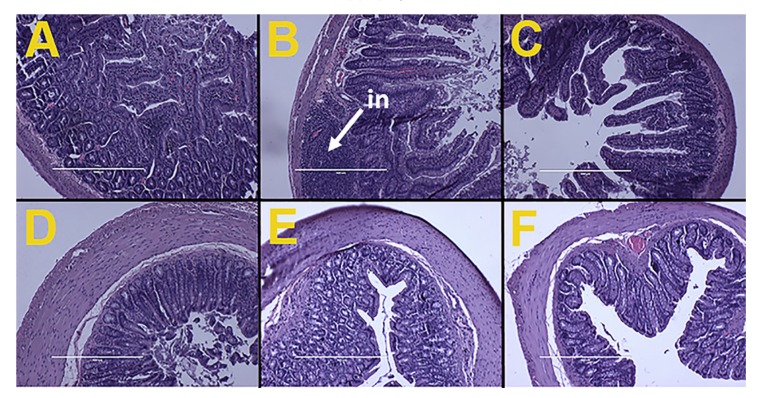
Effects of *Kappaphycus* on structure and inflammation in ileum (top row) and colon (bottom row) using hematoxylin and eosin stain in C rats (**A**,**D**), H rats (**B**,**E**), and HR rats (**C**,**F**). “in”—inflammation.

Ileum from H rats showed infiltration of inflammatory cells while ileum and colon from C and HR rats along with colon from H rats did not show any structural damages, including no evidence of inflammation. This indicated that the *Kappaphycus* did not cause any inflammatory damage to the gut structure (Figure 2).

**Figure 3:**
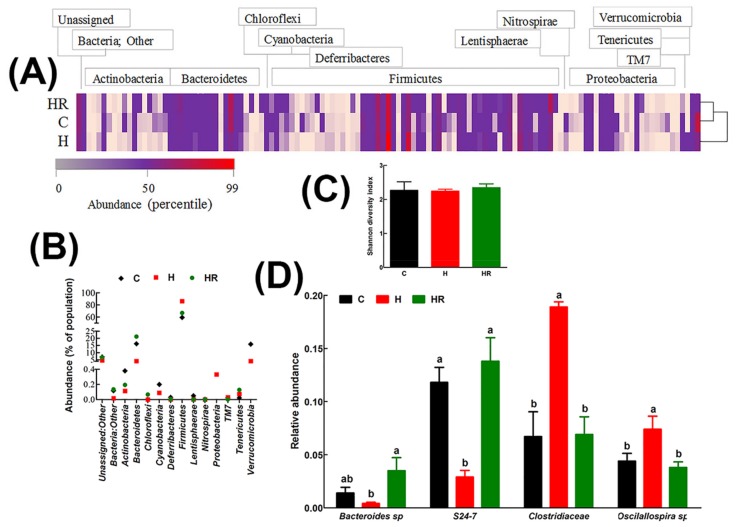
Effect of *Kappaphycus* on gut microbiota diversity profiles. (**A**) Heat map of bacterial species abundance. Abundance values for each species were plotted as a percentile with the most abundant species represented in bright red, the 50th percentile species represented in purple and the lowest value in pink. (**B**) The relative abundance of each phylum presented as a percentage of the total population for each treatment group. (**C**) Shannon diversity index. The index was determined from the means of abundance for each treatment group. (**D**) Relative abundance of species that were differentially regulated by diet. Only species whose mean abundance for the C diet group was equal to or higher than 1% were plotted in order to enhance confidence. C, corn starch diet-fed rats; H, high-carbohydrate, high-fat diet-fed rats; HR, high-carbohydrate, high-fat diet-fed rats supplemented with dried and milled whole *Kappaphycus*.

In all groups, the major gastrointestinal bacterial phyla, Firmicutes and Bacteroidetes, were predominant (Figure 3). Relative to C and HR rats, H rats had lower abundance of Bacteroidetes by 12.05% and 17.04%, respectively. Conversely, H rats had 26.97% and 19.43% more abundance of Firmicutes relative to C and HR rats, respectively (Figure 3B). There was no significant difference in the abundance of both Firmicutes and Bacteroidetes between C and HR rats. Based on the Shannon diversity index, there was no difference in diversity between the groups (Figure 3C).

The relative abundances of all species were compared between the groups to estimate the effect of diet at species level. A cut-off point of 1% abundance in C rats was applied to enhance confidence. The abundance of two species from phylum Bacteroidetes (*Bacteroides* sp., and an unspecified species from the S24-7 family) and 2 species from phylum Firmicutes (*Oscillospira* sp. and an unspecified species from Clostridiaceae family) were differentially affected by diet (Figure 3D). Compared to C rats, there was a 3.5-fold decrease in *Bacteroides* sp. and 4-fold decrease in the S24-7 family species. HR rats had higher abundance of *Bacteroides* sp. 2.5-fold and 8.8-fold relative to C and H rats, respectively. Similarly, HR rats had higher S24-7 family species by 4.8-fold compared to H rats and normalized its abundance to C rats. H rats had higher abundance of *Oscillospira* sp than C and HR rats (1.7 and 1.9-fold higher, respectively). Similarly, H rats had higher abundance of Clostridiaceae family by 2.8 and 2.7-fold, respectively, relative to C and HR rats. There was no difference in both the Firmicutes species between C and HR rats. Some species in phylum Actinobacteria, for example *Bifidobacterium pseudolongum*, were more abundant in C and HR rats than in H rats, but their abundance was below the cut-off of 1%.

## 4. Discussion

We have used an established rat model of human metabolic syndrome to demonstrate the anti-obesity properties of whole *Kappaphycus*. This red seaweed has a high content of *κ*-carrageenan and a low content of digestible carbohydrates coupled with a high potassium (K) content [31]. Foods with a high soluble fiber content improved symptoms of metabolic syndrome by increasing gastrointestinal viscosity thereby inhibiting intestinal absorption of lipids and carbohydrates [8,32,33,34]. Sulfated polysaccharides, including carrageenans, alginates, and porphyrans derived from red seaweeds, and fucoidans derived from brown seaweeds, have shown anti-obesity, blood glucose-lowering, and blood lipid-lowering effects in different experimental models [32,35,36]. In a mouse model of obesity, treatment with 1% fucoidan suppressed adiposity by transcriptionally inhibiting the expression of aP2 and PPAR-*γ* as well as inhibiting acetyl-CoA carboxylase activity, thereby decreasing fatty acid synthesis [36]. In obese individuals, treatment with sodium alginate from the seaweeds *Laminaria hyperborea* and *Laminaria digitata* (Class Phaeophyceae) in a calorie-restricted diet led to increased loss of body weight by 6.4% in a 12-week trial [37].

In this study, treatment with whole *Kappaphycus* decreased central obesity and lowered plasma triglycerides and non-esterified fatty acids. However, it is unlikely that these observed changes were mediated by decreased carbohydrate and lipid absorption due to gastrointestinal viscosity. Lowered fecal lipids suggests that *Kappaphycus* increased lipid catabolism rather than an inhibition of absorption in the gut. It is also possible that, similar to fucoidan, carrageenan attenuated adiposity and dyslipidemia by inhibiting adipogenesis and fatty acid synthesis or increasing lipase activity [36,38,39]. In 3T3-L1 adipocytes, 200 μg/mL fucoidan increased the expression of hormone-sensitive lipase and its phosphorylated form signifying increased lipolysis. Since the decreased triglycerides in our study were accompanied by decreased non-esterified fatty acids, it is possible that, apart from stimulating lipolysis, *Kappaphycus* stimulated fatty acid oxidation as well. Fucoidans potentially increased fatty acid oxidation through stimulation of AMPK activity [40] and carrageenans may have similar AMPK-stimulating properties.

*Kappaphycus*, a tropical red seaweed (formerly *Eucheuma cottonii*), is a commercial source of carrageenan, a soluble fiber and sulfated polysaccharide that is extracted and used as an emulsifier in the food industry [41]. In addition to fiber, *Kappaphycus* contains many minerals including potassium (K), calcium (Ca), magnesium (Mg), sodium (Na), copper (Cu) and zinc (Zn) [42]. Typically, production of *Kappaphycus* occurs in shallow water fixed-line culture on the reef flat, followed by air-drying for a few days adjacent to the farm sites. *Kappaphycus* is normally dried to 25–40% moisture content and then transported to trading centres and distributed to processors for extraction of semi-refined carrageenan through a heated alkali process.

Throughout the tropical countries, there is now a push for diversification of the products from *Kappaphycus*, with a particular emphasis on cottage industry use of the whole seaweed as a vegetable and in savory and sweet products. This change from the traditional industrial extraction of carrageenan to use as a whole food for local consumption brings with it questions as to the potential health benefits of the unrefined products of dried seaweed.

In many countries, there is a renewed push for the use of whole foods in particular since metabolic diseases associated with energy-rich diets are a leading cause of public health concern [43]. Increased energy intake without increased energy expenditure leads to development of central obesity, hyperglycemia, dyslipidemia, fatty liver, hypertension, and insulin resistance, collectively known as metabolic syndrome [44]. Functional foods such as red seaweeds could serve as potential therapeutic options for these changes [12] and there is some evidence that *Kappaphycus* has antioxidant and blood lipid-lowering properties [13,14]. Furthermore, an in vitro study using the extracted sulfated fractions of *κ*-carrageenan from *Kappaphycus* demonstrated the potential of this seaweed as an intervention for colon cancer [15].

Contrary to some reports of adverse gastrointestinal effects of foods containing carrageenans [45], this study did not show changes in the mucosal structure of intestine after feeding *Kappaphycus* to rats. However, the focus to date on the potential effects of carrageenan, both positive and negative, ignores the potential independent and interactive effects of the other main components, in particular the seaweed salt as the major component of *Kappaphycus*.

Phytochemicals from *Kappaphycus* other than carrageenans may confer beneficial effects. For example, phycoerythrin and phycocyanin may stimulate triglyceride catabolism by providing an antioxidant environment [46] which is required for optimal lipase activity [47]. *Kappaphycus* also contains monounsaturated fatty acids and polyunsaturated fatty acids [42,48] which are associated with beneficial changes in metabolic syndrome such as increased HDL-cholesterol, decreased triglycerides, and improved cardiovascular and liver health [49,50]. However, these pigments and fatty acid components are unlikely to be present in sufficient quantities in *Kappaphycus* to produce these changes in metabolic syndrome [32].

Potassium may be present in sufficient amounts in *Kappaphycus* to produce physiological changes. In this study, a lower serum potassium concentration in obese rats was accompanied by pathological changes in the cardiovascular system. Potassium is a key determinant of cardiovascular health with low dietary intake considered a major factor in dysregulation of blood pressure in patients with hypertension and also in normotensive individuals who are at risk of developing cardiovascular disease [51,52]. In a meta-analysis involving 2600 normotensive and hypertensive subjects, a median dose of 75 mmol potassium/day (supplemented through diet) decreased systolic blood pressure by 3.11 mmHg and diastolic blood pressure by 1.97 mmHg [52,53]. The main source of potassium in humans is the diet, especially fresh fruits, vegetables, and milk, accounting for 40–100 mmol daily intake [54]. In 2012, WHO strongly recommended an increase in potassium intake from food to reduce blood pressure and risk of cardiovascular disease, stroke, and coronary heart disease and conditionally recommended a daily potassium intake of at least 90 mmol (3510 mg) for adults [55]. Although it is possible to achieve this target by consuming conventional foods such as rice and vegetables, these foods often have a high energy content or may not be consumable in sufficient amounts to confer the benefit. For example, a daily serving of approximately 300 g of brown rice or 700 g of spinach, two of the most potassium-rich foods, would provide the entire potassium requirements. *Kappaphycus*, with 20% potassium of its dry weight, would meet the same requirement with only ~18 g dried material. Therefore, the use of *Kappaphycus* as a functional food is a feasible intervention due to its high potassium content for individuals who are at risk of cardiovascular disease.

This study has demonstrated positive effects of *Kappaphycus* on gut microbiota, selecting against Firmicutes and promoting Bacteroidetes. In humans and animal models, obesity is associated with increases in the Firmicutes and decline in the Bacteroidetes [56,57]. The positive shift in gut microbiota associated with health benefits of certain diets has been attributed partly to dietary fiber [58,59]. Fiber may increase digesta mass, thereby shortening transit time and increasing defecation frequency as well as through increased fermentation leading to enhanced bacterial proliferation [60]. The increase in *Bacteroides* sp. and S24-7 family species following *Kappaphycus* supplementation may therefore be due to carrageenan. A recent study has shown that *Bacteroides* sp. are able to digest carrageenan [61], producing oligosaccharides which possess lipid-lowering properties [62]. Further, an in vitro study has confirmed the role of carrageenan from *Kappaphycus* as a prebiotic [63], which would give a plausible explanation for the improvements in gut microbiota.

*Bacteroides thetaiotaomi* liberated carbohydrate-degrading enzymes in the gastrointestinal environment which support the growth of other beneficial bacteria such as *Bifidobacterium longum* [64]. Strains of the S24-7 family, also known as *Candidatus* Homeothermaceae, produced short-chain fatty acids including acetate, propionate, and succinate [65], which confer benefits against metabolic syndrome. Further, many species from gut bacteria also produce vitamins in the B family including thiamine, riboflavin, niacin, pantothenate, biotin, and folate [66]. In a batch culture system inoculated with human feces and designed to mimic the distal colon, *Kappaphycus* stimulated the proliferation of bacteria producing short-chain fatty acids, suggesting that the increase in the S24-7 family in this study may be a mechanism for increasing production of short-chain fatty acids [63]. The decrease in *Clostridiaceae* is noteworthy since members of the *Clostridiaceae* family are associated with poor metabolic outcomes [67,68] and strategic targeting of the family is a feasible intervention for diet-induced obesity [69]. Paradoxically, the *Oscillospira* genus which has been positively associated with leanness or lower body mass index in humans [70] was more abundant in obese rats than the *Kappaphycus*-treated rats despite the decrease in body weight in the latter group. This discrepancy may be caused by differences between species as one study with high-fat diet-fed rats suggested that *Oscillospira* in the ileum predisposes the individual to obesity and metabolic disorders [71].

This study treated rats with 5% *Kappaphycus* in food as in our previous studies with other microalgae and seaweeds [8,33]. This dose of seaweed translates to ~30 g daily intake of red seaweed for an adult human [72]; the average daily intake of seaweeds in Japanese people was 14.3 g [73]. The dried red seaweed contains 34.6% of dietary fiber equating to ~10 g of dietary fiber daily in humans. Mean dietary fiber intake of the US population for 1999–2008 was 13.1–16.1 g/day [74]. Further, the American Dietetic Association recommended a daily dietary fiber intake of 25g for adult females and 38 g for adult men [75]. The dietary fiber intake from red seaweed in this study falls within the recommended daily fiber intake when added to mean US intake, also providing more than the recommended potassium intake. Future studies with such seaweeds warrant measurement of short-chain fatty acids that are produced through the supply of dietary fiber. Moreover, measurement of intestinal permeability and plasma concentrations of endotoxins would provide more insight into the role of seaweeds in improving gut structure and function to improve metabolic syndrome.

## 5. Conclusions

This study demonstrated the potential of *Kappaphycus* as a functional food with possible application in the prevention of metabolic syndrome. We have further demonstrated that *Kappaphycus* may reverse metabolic syndrome through selective inhibition of obesogenic gut bacteria and promotion of health-promoting gut bacteria. There were no negative effects of *Kappaphycus* on any of the measured variables.

## Figures and Tables

**Table 1:** Effects of *Kappaphycus* on metabolic, body composition, and physiological variables.

Variables	C	H	HR
Initial body weight, g	331 ± 1	334 ± 1	332 ± 1
Final body weight, g	350 ± 8 ^b^	431 ± 11 ^a^	348 ± 6 ^b^
Body mass index, g/cm^2^	0.57 ± 0.02 ^b^	0.70 ± 0.03 ^a^	0.58 ± 0.01 ^b^
Water intake, mL/day	37.3 ± 7.3	21.7 ± 2.2	35.0 ± 2.6
Food intake, g/day	38.7 ± 3.3 ^a^	23.7 ± 2.3 ^b^	20.8 ± 2.0 ^b^
Energy intake, kJ/day	434 ± 11 ^b^	485 ± 8 ^a^	507 ± 10 ^a^
Feed conversion efficiency, g/kJ	0.04 ± 0.02 ^b^	0.20 ± 0.03 ^a^	0.03 ± 0.01 ^b^
Abdominal circumference, cm	18.4 ± 0.1 ^c^	20.5 ± 0.1 ^a^	18.9 ± 0.1 ^b^
Retroperitoneal fat, mg/mm *	149 ± 14 ^b^	284 ± 31 ^a^	157 ± 26 ^b^
Epididymal fat, mg/mm *	79 ± 10	141 ± 29	81 ± 17
Omental fat, mg/mm *	114 ± 10 ^b^	208 ± 20 ^a^	99 ± 20 ^b^
Total abdominal fat, mg/mm *	342 ± 18 ^b^	632 ± 73 ^a^	337 ± 48 ^b^
Total fat mass, g	49.4 ± 5.0 ^b^	98.7 ± 8.3 ^a^	53.4 ± 6.5 ^b^
Total lean mass, g	315 ± 4 ^a^	318 ± 7 ^a^	290 ± 6 ^b^
Bone mineral content, g	10.1 ± 0.3 ^b^	11.8 ± 0.3 ^a^	9.8 ± 0.2 ^b^
Bone mineral density, g/cm^2^	0.165 ± 0.002 ^b^	0.180 ± 0.005 ^a^	0.156 ± 0.002 ^c^
Basal blood glucose concentrations, mmol/L	3.7 ± 0.3 ^b^	4.7 ± 0.2 ^a^	3.6 ± 0.2 ^b^
Area under the curve, mmol/L·min	650 ± 29 ^b^	799 ± 27 ^a^	753 ± 30 ^a^
Total cholesterol, mmol/L	1.50 ± 0.08	1.61 ± 0.09	1.76 ± 0.14
Triglycerides, mmol/L	0.61 ± 0.09 ^c^	1.65 ± 0.21 ^a^	1.31 ± 0.07 ^b^
Non-esterified fatty acids, mmol/L	1.16 ± 0.21 ^c^	4.09 ± 0.29 ^a^	1.72 ± 0.36 ^b^
Fecal lipids, mg/g of feces	0.87 ± 0.04 ^b^	1.31 ± 0.04 ^a^	0.63 ± 0.03 ^c^
Systolic blood pressure, mmHg	120 ± 2 ^c^	136 ± 1 ^a^	127 ± 3 ^b^
LV + septum wet weight, mg/mm *	17.8 ± 0.6	20.0 ± 0.8	19.2 ± 0.9
RV wet weight, mg/mm *	4.25 ± 0.30	4.08 ± 0.46	4.61 ± 0.34
Liver weight, mg/mm *	217 ± 14	286 ± 9	256 ± 30
Plasma ALT activity, U/L	28.0 ± 3.6 ^b^	30.1 ± 4.6 ^b^	42.0 ± 4.0 ^a^
Plasma AST activity, U/L	70.9 ± 3.5	72.1 ± 7.0	74.0 ± 4.0
Plasma potassium, mmol/L	5.7 ± 0.3 ^a^	5.0 ± 0.3 ^b^	6.1 ± 0.5 ^a^

Values are mean ± SEM, *n* = 8–10. Means in a row with unlike superscripts (a, b, or c) differ and no superscript indicates no significant difference between the groups, *p* < 0.05. ALT, alanine transaminase; AST, aspartate transaminase; C, corn starch diet-fed rats; H, high-carbohydrate, high-fat diet-fed rats; HR, high-carbohydrate, high-fat diet-fed rats supplemented with dried and milled whole *Kappaphycus*. LV, left ventricle; RV, right ventricle. * indicates the values were normalized against tibial length and presented as the tissue weight in mg/mm tibial length.

**Table 2:** Effects of *Kappaphycus* on metal and metalloid contents in liver.

Metal (Symbol)	C (in ppm)	H (in ppm)	HR (in ppm)
Aluminium (Al)	5.32 ± 0.89	11.83 ± 2.71	5.71 ± 1.50
Arsenic (As)	4.75 ± 0.71 ^a^	2.24 ± 0.34 ^b^	1.40 ± 0.16 ^b^
Boron (B)	BDL	BDL	BDL
Barium (Ba)	0.07 ± 0.01 ^b^	0.23 ± 0.04 ^a^	0.07 ± 0.01 ^b^
Calcium (Ca)	124 ± 3 ^a^	117 ± 6 ^a^	94 ± 9 ^b^
Cadmium (Cd)	0.06 (*n* =1)	BDL	0.04 ± 0.01 (*n* = 2)
Cobalt (Co)	BDL	BDL	BDL
Chromium (Cr)	BDL	BDL	BDL
Copper (Cu)	13.78 ± 1.32	10.65 ± 0.99	9.95 ± 1.41
Iron (Fe)	575 ± 22 ^a^	376 ± 12 ^b^	405 ± 38 ^b^
Mercury (Hg)	BDL	BDL	BDL
Potassium (K)	10,506 ± 256 ^a^	10,146 ± 309 ^a^	7225 ± 998 ^b^
Magnesium (Mg)	547 ± 10	529 ± 39	434 ± 40
Manganese (Mn)	4.67 ± 0.20	3.92 ± 0.38	4.41 ± 0.49
Molybdenum (Mo)	0.65 ± 0.07 ^ab^	0.81 ± 0.05 ^a^	0.42 ± 0.09 ^b^
Sodium (Na)	1581 ± 97	1,278 ± 44	1060 ± 187
Nickel (Ni)	0.14 (*n* = 1)	BDL	0.57 ± 0.37 (*n* = 4)
Phosphorus (P)	8820 ± 324 ^a^	8559 ± 399 ^a^	6090 ± 730 ^b^
Lead (Pb)	0.15 ± 0.05	0.11 ± 0.02	0.11 ± 0.03
Sulfur (S)	5233 ± 155 ^a^	5055 ± 239 ^a^	3510 ± 408 ^b^
Selenium (Se)	1.54 ± 0.03	1.45 ± 0.13	1.01 ± 0.16
Strontium (Sr)	0.20 ± 0.02 ^b^	0.31 ± 0.05 ^a^	0.17 ± 0.02 ^b^
Vanadium (V)	BDL	BDL	BDL
Zinc (Zn)	63.12 ± 2.81 ^a^	66.85 ± 2.36 ^a^	43.23 ± 5.13 ^b^

Values are mean ± SEM, *n* = 4–5. Means in a row with unlike superscripts (a or b) differ and no superscript indicates no significant difference between the groups, *p* < 0.05. C, corn starch diet-fed rats; H, high-carbohydrate, high-fat diet-fed rats; HR, high-carbohydrate, high-fat diet-fed rats supplemented with dried and milled whole *Kappaphycus*; BDL, below detection limit.

## Supplementary Materials

The following are available online at www.mdpi.com/2072-6643/9/11/1261/s1, Table S1: *Kappaphycus* amino acid composition; Table S2: *Kappaphycus* CHONPS and halogens; Table S3: *Kappaphycus* Metals and Metalloids.
