# Supplementary material for: Kappaphycus alvarezii as a Food Supplement Prevents Diet-Induced Metabolic Syndrome in Rats
Source: Nutrients. 2017 Nov 17;9(11):1261. doi: 10.3390/nu9111261 (PMC5707733; doi:10.3390/nu9111261)
Supplement: Supplementary file 1 [file nutrients-09-01261-s001.pdf]

nutrients

MDPI

Article

Kappaphycus alvarezii as a Food Supplement Prevents Diet-Induced Metabolic Syndrome in Rats

Stephen Wanyonyi [1], Ryan du Preez [1,2], Lindsay Brown [1,2], Nicholas A. Paul [3] and Sunil K. Panchal [1,*]

[1] Functional Foods Research Group, Institute for Agriculture and the Environment, University of Southern Queensland, Toowoomba, QLD 4350, Australia; Stephen.Wanyonyi@usq.edu.au (S.W.); Ryan.duPreez@usq.edu.au (R.d.P.); Lindsay.Brown@usq.edu.au (L.B.)
[2] School of Health and Wellbeing, University of Southern Queensland, Toowoomba, QLD 4350, Australia
[3] Faculty of Science, Health, Education and Engineering, University of the Sunshine Coast, Maroochydore, QLD 4558, Australia; npaul@usc.edu.au
* Correspondence: Sunil.Panchal@usq.edu.au; Tel.: +61-7-4631-1012

Supplementary Materials:

Supplementary Table S1. *Kappaphycus* amino acid composition

**Biomass properties (% *dry weight*)**	**Mean**	**SD**
Histidine	0.09	0.01
Serine	0.80	0.01
Arginine	0.83	0.01
Glycine	0.80	0.02
Aspartic acid	1.71	0.03
Glutamic acid	1.80	0.03
Threonine	0.84	0.01
Alanine	0.92	0.01
Proline	0.70	0.01
Lysine	0.61	0.01
Tyrosine	0.20	0.01
Methionine	0.21	0.00
Valine	1.06	0.02
Isoleucine	0.79	0.01
Leucine	1.25	0.03
Phenylalanine	0.82	0.01

Values are mean ± SD, $n = 2$.

Supplementary Table S2. *Kappaphycus* CHONPS and halogens

**Biomass properties (% d.w.)**	**Mean**	**SD**
Carbon (C)	14.85	4.96
Hydrogen (H)	2.58	0.91
Oxygen (O)	20.00	4.01
Nitrogen (N)	0.31	0.00
Phosphorus (P)	0.03	0.01
Sulfur (S)	3.11	1.01
Chlorine (Cl)	23.43	7.39
Bromine (Br)	0.12	0.01
Fluorine (F)	0.00	0.00
Iodine (I)	< 0.02	-

Values are mean ± SD, *n* = 2.

Supplementary Table S3. *Kappaphycus* metals and metalloids

**Biomass properties (*mg/kg*)**	**Mean**	**SD**
Aluminum (Al)	108.00	12.73
Arsenic (As)	4.27	0.11
Boron (B)	118.00	5.66
Barium (Ba)	1.27	0.29
Calcium (Ca)	8,964.00	599.63
Cadmium (Cd)	0.07	0.01
Cobalt (Co)	0.64	0.01
Chromium (Cr)	25.30	8.34
Copper (Cu)	1.90	0.97
Iron (Fe)	424.50	7.78
Mercury (Hg)	0.22	0.08
Potassium (K)	200,003.50	3,457.05
Magnessium (Mg)	5,692.50	208.60
Manganese (Mn)	4.67	0.73
Molybdenum (Mo)	<0.10	-
Sodium (Na)	37,692.50	239.71
Nickel (Ni)	7.84	4.19
Lead (Pb)	0.15	0.03
Selenium (Se)	<1.00	-
Strontium (Sr)	147.50	19.09
Vanadium (V)	4.13	0.26
Zinc (Zn)	4.46	-

Values are mean ± SD, *n* = 2.
